# Surgery for colorectal liver metastases: Predictive factors of biliary fistula

**DOI:** 10.1016/j.sopen.2026.01.006

**Published:** 2026-01-31

**Authors:** Mohamed Guelbi, Mohamed Hajri, Zied Hadrich, Aziz Atallah, Sofiene Gabsi, Rached Bayar, Lassad Gharbi, Sahir Omrani

**Affiliations:** a Department of Surgery, Mongi Slim Hospital, Marsa, Tunisia

**Keywords:** Liver, Metastasis, Biliary fistula, Colorectal, Associated factors

## Abstract

**Background:**

Colorectal liver metastases (CRLM) are the most common secondary site of colorectal cancer. Hepatic resection remains the potentially curative standard treatment, but postoperative morbidity remains substantial, with biliary fistula representing the most frequent and clinically significant specific complication. This study aimed to identify predictive factors of biliary fistula following CRLM surgery.

**Methods:**

A retrospective single-center study was conducted including 129 patients who underwent surgery for CRLM at Mongi Slim Hospital, La Marsa, between January 2020 and December 2024. The primary endpoint was postoperative biliary fistula according to ISGLS criteria. Univariate and multivariate logistic regression analyses were performed to determine independent predictive factors.

**Results:**

Postoperative biliary fistulas occurred in 31 patients (24%). They were detected through surgical drainage in 61%, through abdominal collections in 29%, and as biliary peritonitis in 9.7%. Most fistulas were grade A (71%), followed by grades B (16%) and C (13%). Spontaneous resolution occurred in 67.7% of cases, while 19.4% required percutaneous drainage and 12.9% required surgical re-intervention. Univariate analysis identified several factors associated with biliary fistula: low BMI, elevated preoperative PAL and GGT levels, preoperative cholestasis, and sinusoidal obstruction syndrome. In multivariate analysis, three independent predictors were retained: low BMI (OR = 0.818; *p* = 0.04), postoperative hyperleukocytosis (OR = 4.001; *p* = 0.028), and postoperative cholestasis (OR = 8.382; *p* = 0.041). Overall postoperative morbidity reached 43.4%, with no postoperative mortality.

**Conclusion:**

Biliary fistula remains a major complication after CRLM resection. Identifying high-risk patients may improve postoperative surveillance and outcomes.

## Highlights

• Postoperative biliary fistula occurred in 24% of patients after CRLM resection, with most cases classified as low-grade
• Lower BMI, postoperative hyperleukocytosis, and postoperative cholestasis were independent predictors of biliary fistula
• Biological and nutritional assessment may improve risk stratification and postoperative surveillance in CRLM surgery

Colorectal cancer is among the most frequent malignancies worldwide and remains a leading cause of cancer-related mortality [1]. The liver represents the predominant site of distant spread, with up to 20% of patients presenting with synchronous colorectal liver metastases at diagnosis and more than half developing liver involvement during the course of their disease [2]. Hepatic resection remains the cornerstone of curative treatment for resectable colorectal liver metastases and offers the best chance of long-term survival, with reported five-year survival rates approaching 60% in specialized centers. Despite continuous progress in surgical techniques, perioperative care and oncologic management, postoperative morbidity after hepatectomy remains substantial. Among procedure-specific complications, biliary fistula represents a particularly relevant source of morbidity, leading to intra-abdominal sepsis, prolonged drainage, delayed chemotherapy, repeated interventions and extended hospitalization. Although its clinical impact is well recognized, biliary fistula has been less systematically investigated than other complications such as liver failure or infectious events, and data focusing specifically on patients undergoing resection for colorectal liver metastases remain limited.

Most available studies have analyzed heterogeneous populations including hepatocellular carcinoma and benign liver disease, or have focused on overall morbidity rather than biliary complications. Furthermore, earlier reports frequently predated the standardized definition proposed by the International Study Group of Liver Surgery, which has improved the consistency of diagnosis and reporting [3]. Identifying reliable predictive factors of biliary fistula in the setting of colorectal liver metastases is therefore essential to optimize perioperative decision-making and postoperative surveillance. The present study aimed to assess the incidence, clinical presentation, severity and management of biliary fistula after hepatic resection for colorectal liver metastases and to identify independent predictive factors that may guide risk stratification.

This retrospective observational study included all adult patients who underwent hepatic resection for histologically confirmed colorectal liver metastases in a single hepatobiliary unit between January 2020 and December 2024. Patients were included irrespective of the timing or number of metastases and of the extent of liver resection, and were excluded only if biliary reconstruction had been performed. Postoperative biliary fistula was defined according to International Study Group of Liver Surgery criteria as the presence of bile in surgical or radiological drains or identification of bile during re-intervention within 30 postoperative days, and was graded as A, B or C according to severity [3]. Demographic data, comorbidities, tumor characteristics, preoperative biological parameters, neoadjuvant chemotherapy, operative details, pathological findings and postoperative outcomes were collected. Statistical analyses were performed using univariate and multivariate logistic regression models to identify independent predictors of biliary fistula.

**Fig. 1:**
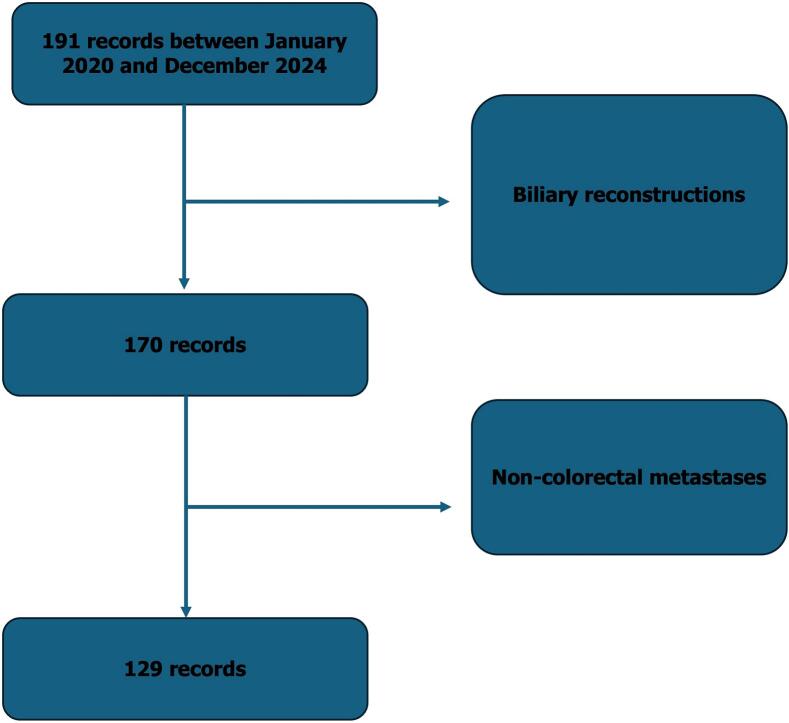
Flow chart of patient selection for the study.

A total of 129 consecutive patients were included (Fig. 1). The mean age was 60.7 years and a slight male predominance was observed with a sex ratio of 1,26. Overweight and obesity were present in more than half of patients and comorbidities were frequent. Metastases were synchronous in 53.5% of cases and bilobar in 63.7%, and neoadjuvant chemotherapy was administered in 82.9%, often including oxaliplatin-based regimens. Parenchymal-sparing resections predominated, with metastasectomy (65.1%) and segmentectomy (20.9%) representing the majority of procedures, while major hepatectomy accounted for 22.5%. Postoperative complications occurred in 43.4% of patients and procedure-specific complications in 38.8%. Biliary fistula was diagnosed in 31 patients, corresponding to an incidence of 24%. Most fistulas were low-grade, with 71% classified as grade A, and were diagnosed early through systematic drainage. Spontaneous closure occurred in two-thirds of cases, while percutaneous drainage and surgical re-intervention were required in 19.4% and 12.9%, respectively.

In univariate analysis, lower body mass index, preoperative cholestatic abnormalities, sinusoidal obstruction syndrome, postoperative hyperleukocytosis and postoperative cholestasis were significantly associated with biliary fistula. Multivariate analysis identified three independent predictors: lower body mass index, postoperative hyperleukocytosis and postoperative cholestasis. Tumor-related variables, extent of resection and neoadjuvant chemotherapy were not independently associated with bile leakage.

The incidence of biliary fistula observed in the present study lies in the upper range of published data, which generally vary between 6% and 27% after hepatectomy for colorectal liver metastases [4]. This elevated incidence may be partly explained by systematic drainage allowing early detection of low-grade fistulas and by the high prevalence of neoadjuvant chemotherapy, which is known to induce parenchymal and biliary injury [5]. The predominance of grade A fistulas and the favorable rate of spontaneous closure are consistent with previous reports and confirm that most bile leaks can be managed conservatively when detected early [6].

The identification of low body mass index as an independent predictor is noteworthy. While obesity has traditionally been associated with increased postoperative morbidity, recent evidence suggests that low body mass index may reflect impaired nutritional status and tissue fragility, predisposing to biliary complications [7]. This finding highlights the importance of preoperative nutritional assessment and optimization in patients scheduled for hepatectomy. Postoperative cholestasis emerged as the strongest independent predictor of biliary fistula, supporting the hypothesis that impaired biliary excretion compromises hepatic regeneration and promotes bile leakage along transection surfaces [4]. Likewise, postoperative hyperleukocytosis likely reflects an early inflammatory response to bile leakage or subclinical infection and may represent a useful biological warning signal, as previously suggested by other authors [8].

Sinusoidal obstruction syndrome was significantly associated with biliary fistula in univariate analysis. Oxaliplatin-induced sinusoidal injury has been widely documented and is known to increase postoperative morbidity by altering microvascular architecture and biliary integrity [9]. Although neoadjuvant chemotherapy was not independently predictive in multivariate analysis, its indirect contribution through parenchymal injury remains probable.

Several limitations should be acknowledged. The retrospective design and the monocentric setting limit external validity and may introduce selection bias. The sample size may have limited the detection of less frequent predictors, and detailed information on chemotherapy regimens and cumulative doses was unavailable.

Despite these limitations, the present study provides clinically relevant data focusing specifically on biliary fistula after resection for colorectal liver metastases. Our findings emphasize the importance of integrating nutritional evaluation into preoperative assessment and of reinforcing early postoperative biological monitoring, particularly of white blood cell count and cholestatic enzymes, to allow prompt detection of subclinical bile leakage. Future prospective multicenter studies are warranted to validate these predictors and to develop reliable risk-stratification models that may guide perioperative management and reduce the incidence and severity of biliary fistula in this population.

## CRediT authorship contribution statement

**Mohamed Guelbi:** Writing – original draft, Conceptualization. **Mohamed Hajri:** Supervision. **Zied Hadrich:** Writing – original draft. **Aziz Atallah:** Writing – original draft. **Sofiene Gabsi:** Visualization. **Rached Bayar:** Supervision. **Lassad Gharbi:** Writing – review & editing, Validation. **Sahir Omrani:** Writing – review & editing, Validation.

## Consent for publication

Written informed consent was obtained from legal authorized representatives before the study. On request, a copy of the written consent is available for review by the Editor-in-Chief of this journal.

## Ethical approval

Ethical approval is exempted/waived at our institution.

## Funding sources

This work is not funded.

## Declaration of competing interest

The authors declare that they have no known competing financial interests or personal relationships that could have appeared to influence the work reported in this paper.

## Data Availability

This published article includes all the required data.
